# Mitochondrial toxicity and caspase activation in HIV pregnant women

**DOI:** 10.1111/jcmm.12935

**Published:** 2016-08-30

**Authors:** Sandra Hernandez, Constanza Moren, Marc Catalán‐García, Marta Lopez, Mariona Guitart‐Mampel, Oriol Coll, Laura Garcia, Jose Milisenda, Angela Justamante, Josep Maria Gatell, Francesc Cardellach, Eduard Gratacos, Òscar Miro, Gloria Garrabou

**Affiliations:** ^1^ Maternal‐Fetal Medicine DepartmentClinical Institute of Gynecology, Obstetrics and NeonatologyHospital Clinic of BarcelonaBarcelonaSpain; ^2^ Muscle Research and Mitochondrial Function LaboratoryCellex‐IDIBAPSFaculty of Medicine‐University of BarcelonaHospital Clinic of BarcelonaBarcelonaSpain; ^3^ Centro de Investigación Biomédica en Red de Enfermedades Raras (CIBERER)MadridSpain; ^4^ Clinica EuginBarcelonaSpain; ^5^ Infectious DiseaseHospital Clinic of BarcelonaBarcelonaSpain

**Keywords:** HIV, pregnancy, HAART, mitochondrial toxicity, perinatal outcome

## Abstract

To assess the impact of HIV‐infection and highly active anti‐retroviral treatment in mitochondria and apoptotic activation of caspases during pregnancy and their association with adverse perinatal outcome. Changes of mitochondrial parameters and apoptotic caspase activation in maternal peripheral blood mononuclear cells were compared at first trimester of pregnancy and delivery in 27 HIV‐infected and ‐treated pregnant women *versus* 24 uninfected pregnant controls. We correlated immunovirological, therapeutic and perinatal outcome with experimental findings: mitochondrial DNA (mtDNA) content, mitochondrial protein synthesis, mitochondrial function and apoptotic caspase activation. The HIV pregnancies showed increased adverse perinatal outcome (OR: 4.81 [1.14–20.16]; *P* < 0.05) and decreased mtDNA content (42.66 ± 5.94%, *P* < 0.01) compared to controls, even higher in naïve participants. This depletion caused a correlated decrease in mitochondrial protein synthesis (12.82 ± 5.73%, *P* < 0.01) and function (20.50 ± 10.14%, *P* < 0.001), not observed in controls. Along pregnancy, apoptotic caspase‐3 activation increased 63.64 ± 45.45% in controls (*P* < 0.001) and 100.00 ± 47.37% in HIV‐pregnancies (*P* < 0.001), in correlation with longer exposure to nucleoside analogues. HIV‐infected women showed increased obstetric problems and declined genetic and functional mitochondrial parameters during pregnancy, especially those firstly exposed to anti‐retrovirals. The apoptotic activation of caspases along pregnancy is emphasized in HIV pregnancies promoted by nucleoside analogues. However, we could not demonstrate direct mitochondrial or apoptotic implication in adverse obstetric outcome probably because of the reduced sample size.

## Introduction

The effectiveness of highly active antiretroviral treatment (HAART) regimens in reducing mother‐to‐child vertical transmission (MTCT) of HIV‐infection and in delaying disease progression has been demonstrated and should therefore be offered to all pregnancies 1, 2, 3. Anti‐retroviral (ARV) treatment comprised of two nucleoside reverse transcriptase inhibitors (NRTIs) and non‐nucleoside reverse transcriptase inhibitor (NNRTI) or protease inhibitor (PI) is recommended in pregnancy by the United States, World Health Organization and European guidelines 4, 5. Additional measures including selective caesarean or avoidance of breastfeeding are strongly recommended. However, adverse pregnancy outcome have been increasingly reported by several observational studies in HIV‐infected women exposed to HAART 6, 7, 8, 9, 10, 11. Anti‐retrovirals and HIV‐infection have been associated with pre‐eclampsia, stillbirth, pre‐term labour, low birth weight and intrauterine growth restriction (IUGR) 12, 13, 14, 15, 16, 17.

One of the most serious complications associated with ARVs is mitochondrial toxicity. Mitochondrial‐derived clinical effects of NRTI have been firmly established in HIV‐infected non‐pregnant adults 18, 19, 20. These negative effects depend on the capacity of NRTIs to inhibit DNA polymerase gamma, the enzyme devoted to mitochondrial DNA (mtDNA) replication, leading to a decrease in mtDNA copy number and quality, which may, finally, cause mitochondrial dysfunction 18. Depletion of mtDNA has been extensively described in different tissues of human and animal models (placenta, foetal cord blood, heart, adipose tissue, skeletal muscle, brain and kidney, among others) leading to mitochondrial morphologic, metabolic and energetic abnormalities 21, 22, 23, 24, 25. Such mitochondrial disturbances are enhanced by HIV, which has been additionally blamed for triggering apoptosis 26. Associated clinical syndromes have been expanded to include lipoatrophy, peripheral neuropathy, cardiomyopathy, lactic acidosis and hepatic steatosis 27, 28. Moreover, accelerated ‘mitochondrial aging’ associated with ARV may contribute to cardiovascular disease, malignancies and frailty 29.

Maternal death has been described as a result of lactic acidosis in women receiving long‐term treatment with a combination of NRTI 30, 31, especially in the third trimester of pregnancy and in ARV schedules including two NRTIs. Fortunately, severe maternal mitochondrial toxicity associated with NRTI during pregnancy appears to be rare and is reversible on treatment discontinuation. However, milder forms of mitochondrial toxicity are commonly reported and may have future long‐term effects.

Although ARV use during pregnancy is considered safe, data on ARV and pregnancy, especially in HAART, are insufficient, and safety and long‐term health consequences are currently unknown. The few studies on long‐term HAART exposure have focused on foetal and perinatal ARV effects 32, 33, 34, 35, 36, 37, but rarely on maternal‐related problems which may, in turn, affect children.

We recently described that HAART toxicity may cause subclinical mitochondrial damage in pregnant women and their newborn 38 by reducing mtDNA levels, mitochondrial protein synthesis and mitochondrial function. Additionally, increased apoptosis through caspase‐3 activation was observed in HIV‐pregnant women, but not in their children, cross‐sectionally, at delivery.

The aim of this study was to investigate the impact of HIV‐infection and HAART on mitochondria and apoptotic caspase activation during pregnancy to assess their implication in the increase of adverse perinatal outcome characteristic of HIV‐pregnancies to establish potential prenatal prognosis markers.

## Materials and methods

### Design

We performed a single‐site, controlled observational study without intervention to determine longitudinal mitochondrial toxicity and apoptotic caspase activation (from the first trimester of gestation to delivery) in maternal peripheral blood mononuclear cells (PBMC) of HIV‐infected pregnancies compared to uninfected controls.

### Study population

Twenty‐seven asymptomatic HIV‐1‐infected and twenty‐four uninfected women were consecutively included during routine prenatal care at first trimester of gestation in the Hospital Clinic of Barcelona (Barcelona, Spain).

Controls and cases were age and parity matched. The inclusion criteria for pregnant women were: >18 years of age, single pregnancy, delivery >22 weeks of gestation and, for HIV‐patients, previous diagnosis of HIV‐infection.

Patients taking potentially toxic drugs for mitochondria and with familial history of mitochondrial disease were excluded.

The Ethical Committee of our hospital approved the study and it was performed following the Declaration of Helsinki. All participants provided written consent.

### Clinical results

A database was created to collect epidemiological, immunovirological, therapeutic, obstetric, perinatal and experimental data.

Maternal epidemiological parameters included information on maternal age, race and illegal substance abuse.

Immunovirological parameters for HIV‐infected women consisted in quantifying comorbidity with HCV infection, plasmatic HIV‐viral load (by rtPCR), CD4^+^ T‐cell count (by flow cytometry) and time from HIV infection to delivery.

Therapy was administered to all HIV‐pregnant women following international guidelines. HIV‐infected women were stratified according to ARV use during pregnancy. Women naïve for ARV before pregnancy started HAART (double‐NRTI schedule and either one PI or NNRTI drug) during the second trimester of gestation to prevent MTCT.

Information regarding obstetric and perinatal outcome included: parity, mode of delivery, gestational diabetes mellitus, pre‐eclampsia (new onset of hypertension of >140 mmHg systolic or >90 mmHg diastolic pressure and >300 mg proteins/24 hrs urine after 20 weeks of gestation), foetal death (>22 weeks of pregnancy), gestational age at delivery, pre‐term birth (<37 weeks of gestation), birth weight, small newborn for gestational age (<10th percentile), 5‐min Apgar score <7, neonatal admission to intensive care unit and global adverse perinatal outcome.

Finally, experimental data included maternal measures of mitochondrial and apoptotic caspase activation at first trimester of gestation and delivery.

### Sample collection and processing

At the first trimester of pregnancy and immediately after delivery, 20 ml of peripheral blood was collected in EDTA tubes to isolate PBMC by Ficoll gradient and stored at −80°C until analysis. Protein content was measured by Bradford protein dye binding‐based method 39.

### Mitochondrial studies in maternal PBMC

#### Mitochondrial DNA quantification

Total DNA was extracted by phenol‐chloroform procedure. A fragment of mitochondrial DNA‐encoded ND2 and nuclear DNA‐encoded 18SrRNA genes were amplified separately in triplicate by quantitative rtPCR using the Roche Lightcycler‐thermocycler 20. Mitochondrial DNA content was expressed as the ratio between mitochondrial and nuclear DNA amount (ND2mtDNA/18SrRNA nDNA content).

#### Mitochondrial protein synthesis

We performed Western blotting of 20 μg total cell protein through 7/13% SDS‐PAGE and posterior immunoquantification of the mitochondrial DNA‐encoded and located COXII subunit (25.6 kD) with respect to the nuclear DNA‐encoded and mitochondrially located COXIV subunit (15 kD) to compare relative mitochondrial to nuclear protein synthesis (COXII/COXIV) 20.

#### Mitochondrial enzymatic function

Mitochondrial respiratory chain complex II+III (CII+III) enzymatic activities were measured by spectrophotometry according to Rustin *et al*. 37, 40 by following the increase in absorbance at 550 nm of reduced cytochrome c generation (complex III product) after succinate addition (complex II substrate). Specific enzymatic activities were expressed as nanomols of product per minute and milligram of protein (nmols/min.mg prot).

### Apoptotic caspase activation studies

We performed Western blotting of 20 μg total cell protein by 7/13% SDS‐PAGE and posterior immunoanalysis of active (cleaved) caspase‐3 pro‐apoptotic protein expression (17–19 kD) normalized by the content of β‐actin (47 kD) as a cell loading control. Results were expressed as caspase‐3/β‐actin relative content and were interpreted as a marker of advanced apoptotic events 41.

Additionally, caspase‐9 enzymatic activity was measured by means of the luminescent assay Caspase‐Glo^®^ Assay (Ref: G8210, Promega Corporation, Madison, WI 53711 USA) using 20 μg of PBMCs’ protein. Briefly, 50 μl of diluted sample and 50 μl of kit Caspase‐Glo^®^ reagent were mixed in an opaque reading multiwell plate following manufacturer's instructions. After 36 min. of incubation, the plate was read in a Modulus^™^ II Microplate Multimode Reader and caspase‐ 9 activity was calculated based on relative luminescent units normalized to sample protein amount.

### Statistical analysis

Clinical and epidemiological parameters were expressed as means and range interval and experimental results as means and S.E.M. or as a percentage of increase/decrease at delivery compared to first trimester of pregnancy.

Adverse perinatal outcome, mitochondrial and apoptotic caspase activation results of HIV‐infected women were compared to those of uninfected controls to assess the impact of HIV and/or ARV. Additionally, different correlations were sought between: molecular and functional mitochondrial parameters (to ascertain dependence of mitochondrial function on mitochondrial genome) and clinical and experimental data (to assess mitochondrial or apoptotic basis of obstetric and perinatal outcome).

Nonparametric tests were used to determine: case–control differences (Mann–Whitney independent sample analysis), odds ratio (chi‐squared test) and parameter correlation (Spearman's rank coefficient). Significance was set at 0.05.

## Results

### Clinical data

Table 1 shows epidemiological characteristics of the participants and immunovirological and therapeutic data of HIV mothers. All pregnant women were Caucasian, ranging 25–42 years. Non‐significant differences were observed in maternal clinical data between HIV‐positive and HIV‐negative women.

**Table 1:** Epidemiologic, immunovirologic and therapeutic characteristics of HIV‐infected and uninfected pregnant women

	HIV‐positive (*n* = 27)	HIV‐negative (*n* = 24)	*P*
Maternal age at delivery^a^	34.7 (27–42)	33.6 (25–41)	NS
Illegal drug use, *N* (%)	0	0	–
Alcohol use, *N* (%)	0	0	–
HCV infection, *N* (%)	3 (11.1)	1 (4)	NS
HIV RNA copies per ml at delivery^a^	62.3 (49–250)	–	–
CD4 T‐cell count per ml at delivery^a^	560.2 (97–1242)	–	–
Time from diagnosis of HIV infection to delivery (months)^a^	84 (4–228)	–	–
NRTI before pregnancy (months)^a^	48 (0–106)	–	–
NNRTI before pregnancy (months)^a^	3 (0–86)	–	–
PI before pregnancy (months)^a^	12 (0–97)	–	–
NAÏVE (HAART 2nd–3rdtrimesters), *N* (%)	4 (15)	–	–
HAART all trimesters, *N* (%)	23 (85)	–	–
2 NRTI+1 PI, *N* (%)	15 (55.5)		
2 NRTI+1 NNRTI, *N* (%)	8 (29.5)		

a Data are presented as means and range interval.

HCV: hepatitis C virus; HIV: human immunodeficiency virus; HAART: highly active antiretroviral treatment; NRTI: nucleoside‐analogue reverse transcriptase inhibitor; NNRTI: non‐nucleoside analogue reverse transcriptase inhibitor; NS: not significant; PI: protease inhibitors; RNA: ribonucleic acid; *N*: number.

Most HIV‐infected women were under HAART before pregnancy (85%) and only four cases (15%) were ARV‐naïve and started HAART at the second trimester of gestation. Highly active ARV treatment was given to all patients at delivery to avoid MTCT, consisting of two NRTI and either PI (55.5%) or NNRTI (29.5%). The mean time of HIV‐infection and HAART treatment prior to delivery were 84 and 48 months respectively. At delivery, all women had undetectable viral load and received at least 6 months of double‐NRTI treatment. Neither patients nor controls presented clinical manifestations of mitochondrial toxicity.

### Perinatal outcome

Table 2 shows obstetric and neonatal outcome of the study cohort. All infants were HIV‐uninfected with no clinical symptoms of mitochondrial toxicity, and all received 6‐week zidovudine chemoprophylaxis to prevent MTCT. These pregnancies showed a trend to increased gestational diabetes (7.4% *versus* 4.0%), decreased gestational age at delivery (37.5 *versus* 38.6), pre‐term birth (25.9% *versus* 8.0%), reduced birth weight (2879 g *versus* 3170 g), small newborn for gestational age (22.2% *versus* 4.0%) and intensive care unit admission (11.1% *versus* 4.0%). However, only global adverse perinatal outcome (pre‐term birth and small for gestational age events) were significantly increased among HIV‐positive pregnancies [40.7% *versus* 12.0%, OR: 4.81 (1.14–20.6); *P* < 0.05].

**Table 2:** Obstetric and neonatal outcome of the study cohorts

	HIV positive (*n* = 27)	HIV negative (*n* = 24)	OR (95% CI)
Gestational diabetes mellitus, *N* (%)	2 (7.4)	1 (4)	1.84 (0.15–21.7)
Pre‐eclampsia, *N* (%)	0	0	–
Foetal death, *N* (%)	0	0	–
Gestational age at delivery (weeks)^a^	37.5 (32.2–41.2)	38.6 (38.3–40.3)	*P* = NS
Preterm birth (<37 weeks of gestation), *N* (%)	7 (25.9)	2 (8)	3.85 (0.71–20.7)
Birth weight (g)^a^	2879 (1940–4040)	3170 (3130–3320)	*P* = NS
Small newborn for gestational age (<10th percentile), *N* (%)	6 (22.2)	1 (4)	6.51 (0.72–59.19)
5‐min Apgar score <7, *N* (%)	0	0	–
Neonatal intensive care unit admission, *N* (%)	3 (11.1)	1 (4)	2.87 (0.28–29.67)
Global adverse perinatal outcome, *N* (%)	11 (40.7)	3 (12)	4.81 (1.14–20.16) *P* < 0.05

a Data are presented as means and range interval.

95% CI: 95% confidence interval of the mean; *N*: number; NS: not significant; OR: odds ratio.

### Mitochondrial and apoptotic maternal PBMC analysis

Table S1 shows raw data and statistics of all tested experimental parameters.

### MtDNA content

A highly significant progressive reduction of PBMC mtDNA content was observed in HIV pregnancies (42.66 ± 5.94%, *P* < 0.01), not observed in uninfected pregnant controls (18.34 ± 11.73%, *P* = NS), being even greater in naïve HIV‐infected pregnant women (50%, *P* = NS; data not shown).

### Mitochondrial protein synthesis and function

**Figure 1:**
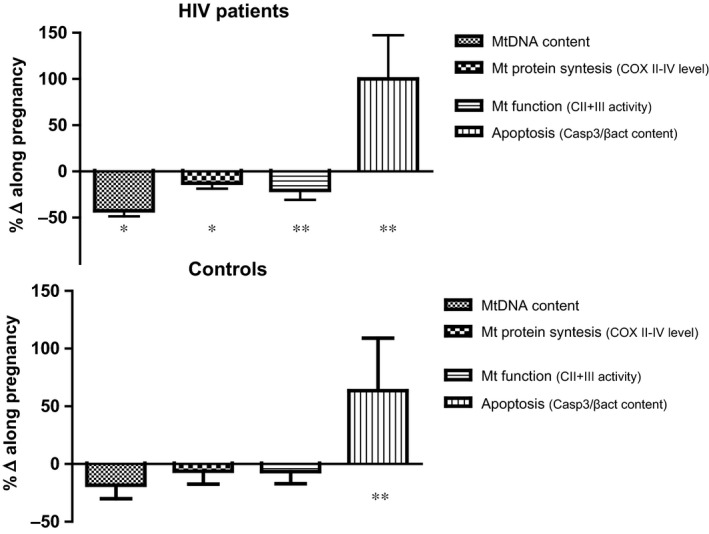
Mitochondrial parameters and apoptotic caspase‐3 activation. Percentage of increase/decrease from first trimester to delivery of mitochondrial parameters or apoptotic caspase‐3 activation in HIV participants and uninfected controls along pregnancy. MtDNA: mitochondrial DNA; COX‐II/IV: Mitochondrial protein synthesis; CII+III: mitochondrial complex II+ complex III enzymatic activity; caspase‐3/β‐Actin: apoptotic caspase‐3 activation. *P < 0.01/**P < 0.001.

Along pregnancy, HIV women also showed a significant decrease in the mitochondrial protein synthesis rate and function, not observed in controls (Fig. 1).

The protein synthesis (COXII/IV expression ratio; Fig. S1) dropped 12.82 ± 5.73% in HIV mothers (*P* < 0.01) but only 6.25 ± 11.25% in controls (*P* = NS), and mitochondrial respiratory chain activity of complex II+III significantly decreased in HIV‐infected mothers (20.50 ± 10.14%, *P* < 0.001), but not significantly in controls (6.64 ± 10.39%, *P* = NS).

### Apoptotic caspase activation

Along pregnancy, HIV‐infected pregnant women and healthy controls presented a marked and significant increase in apoptotic caspase‐3 activation of 100.00 ± 47.37% and 63.64 ± 45.45%, respectively, with respect to baseline (*P* < 0.001 in both cases) (Fig. 1 and Fig. S1). This difference in the apoptotic caspase‐3 activation between cases and controls was significant (*P* < 0.05). Similar findings were observed in the measurement of Caspase‐9 enzymatic activity by means of apoptotic increase along pregnancy in both cohorts of HIV‐infected women and uninfected controls (88.38 ± 41.88 *versus* 15.76 ± 36.83, respectively, *P* = NS), higher for HIV patients (data not shown).

### Associations between molecular and clinical parameters

#### Genetic and functional mitochondrial parameters

**Figure 2:**
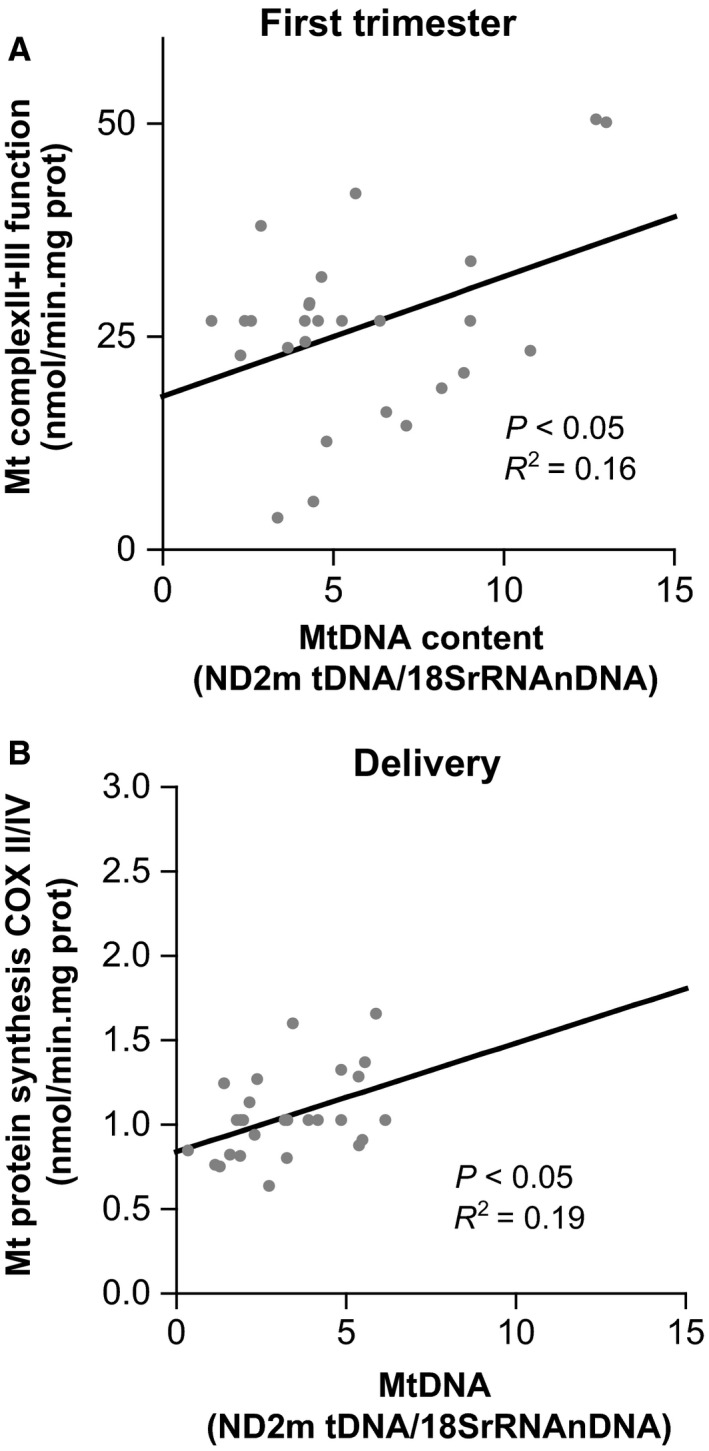
Associations between genetic and functional mitochondrial parameters in HIV women on HAART. (**A**) Association between mitochondrial complex II+III function and mitochondrial DNA content at first trimester. (**B**) Association between mitochondrial protein synthesis COXII/IV and mitochondrial DNA at time of delivery.

In treated HIV‐infected pregnant women, the mitochondrial genome content was positively and significantly correlated with mitochondrial function measured as CII+CIII enzymatic activity in the first trimester of gestation (*P* < 0.05, *R* ^2^ = 0.16; Fig. 2A).

The mitochondrial genome level was also positively and significantly correlated with mitochondrial protein synthesis, by COXII/IV measurement, in HIV‐positive pregnancies at delivery (*P* < 0.05; *R* ^2^ = 0.19; Fig. 2B).

#### Mitochondrial and apoptotic parameters and immunovirological and therapeutic features

**Figure 3:**
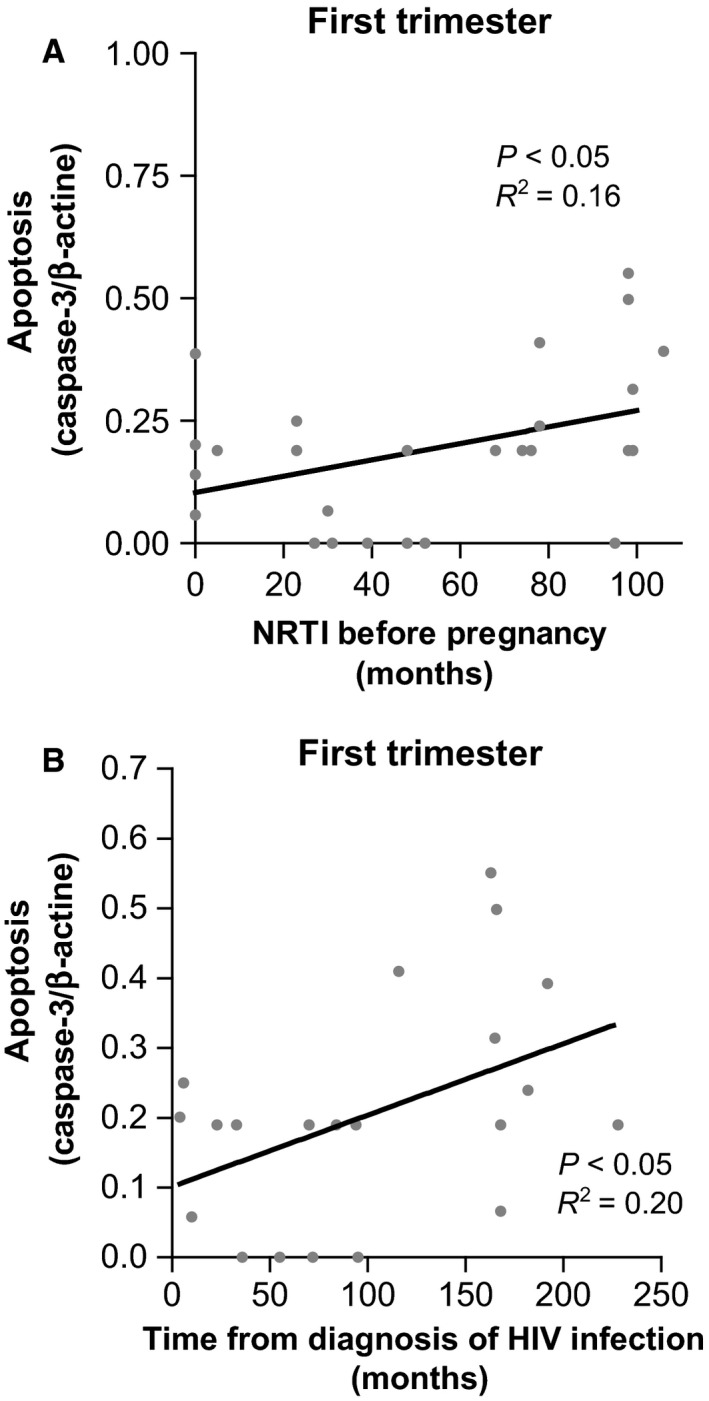
Association between mitochondrial parameters or apoptotic caspase‐3 activation and inmunovirologic and therapeutic characteristics of HIV women on HAART. (**A**) Association between apoptotic caspase‐3 activation and months using NRTI prior to pregnancy. (**B**) Association between apoptotic caspase‐3 activation and months of HIV‐infection.

A significant, positive correlation was found between increased caspase‐3 activation and longer exposure to NRTI prior to pregnancy, in the first trimester of gestation (*P* < 0.05; *R* ^2^ = 0.16; Fig. 3A) and at delivery (data not shown).

A significant, positive correlation was also found between increased caspase‐3 activation in the first trimester and longer HIV infection (*P* < 0.05; *R* ^2^ = 0.20; Fig. 3B).

No correlations were found between mitochondrial maternal experimental parameters and immunovirological status.

#### Mitochondrial and apoptotic parameters and obstetrics results

The global adverse perinatal outcome were significantly increased in HIV pregnancies, but did not correlate with mitochondrial or apoptotic findings.

## Discussion

Anti‐retrovirals are indispensable in the treatment and prevention of HIV infection. Although their use during pregnancy is considered safe, there are still lingering concerns about long‐term health consequences.

Several studies have demonstrated mitochondrial toxicity in animal models, HIV‐infected infants and adults on NRTI therapy and newborns exposed *in utero* to ARVs 22, 24, 37, 38, 47, but it is currently unknown whether HIV‐pregnancy may be an additional risk for the onset of mitochondrial toxicity.

Several physiopathological mechanisms have been proposed to explain the adverse clinical effects in HIV pregnancies. Mitochondrial bioenergetics conditions foetal growth and early postnatal adaptation. Given that mitochondria are exclusively inherited from the maternal ovum, early exposure of the ova and the mitochondria to ARV affects foetal development. We have demonstrated that oocytes from infertile HIV‐infected HAART‐treated women show a decreased mtDNA content that could explain their poor reproductive outcome 19. Several mitochondrial alterations are associated with initiation of mitochondrial biogenesis and activation during early states of embryo development. The mtDNA content and expression levels of genes involved in the maintenance and regulation of biogenesis change during human foetal development. The foetus is exposed to the potentially stressful *in utero* environment created by maternal ARV‐associated metabolic toxicities and placental transferred ARVs at critical points in its development.

Colleoni *et al*. have recently described significantly decreased mtDNA in non‐infected women carrying IUGR fetuses compared to control pregnancies. The authors concluded that in HIV‐uninfected pregnancies mtDNA content and mitochondrial function may help recognizing adverse perinatal outcome 42. It is unknown whether mitochondrial depletion is a predictor factor for poor pregnancy outcome in HIV patients. Indeed, only two studies have evaluated the association between mitochondrial toxicity and poor pregnancy outcome in HIV‐infected pregnant women on HAART. Both analyse mitochondrial status in the third trimester of gestation. Nasi *et al*. described a decrease in mtDNA content in subcutaneous fat of HIV‐pregnant women taking ARV compared to uninfected women without ARV 43. The second report, published by our group, described that HAART toxicity may cause subclinical mitochondrial damage in blood of HIV‐pregnant women and their newborn compared to uninfected pregnancies 27. To our knowledge, the present work is the first longitudinal study investigating the evolution of mtDNA content, mitochondrial protein synthesis, mitochondrial function and apoptotic caspase activation in HIV‐infected women during pregnancy or the potential use of these parameters as pronostics factors to predict adverse perinatal outcome in HIV pregnancies.

In this study we found a higher prevalence of adverse perinatal outcome in the HIV cohort compared with controls, thereby confirming the deleterious effect of both the virus and the treatment on foetal development as well as validating our sample.

During pregnancy, we observed a progressive decrease in mtDNA content which was significantly higher in HIV‐infected pregnant women. Our results agree with two previous studies performed in pregnant women which evaluated blood mtDNA content along pregnancy 38, 39, 40, 41, 42. Colleoni *et al*. reported a significant decrease in mtDNA content in the blood of uninfected pregnant women in the first, second and third trimesters compared to non‐pregnant women 42. This decrease was higher in HIV‐pregnancies 38 and, according to the present findings, would be partially developed along pregnancy.

The mtDNA depletion observed in our HIV cohort was even greater in naïve pregnant women who started HAART during the second trimester of gestation compared with pregnancies under HAART prior to conception. This finding could be explained by the initiation of ARV, abruptly increasing mitochondrial toxicity and resulting in a dramatic decrease in mtDNA content which is maintained over time. This theory is in agreement with a previously published study by our group 44.

In our study we have demonstrated that a decrease in mtDNA leads to a significant reduction in downstream mitochondrial protein synthesis and mitochondrial function in pregnancies complicated with HIV infection and ARV. Correlations were found between genetic and functional mitochondrial parameters demonstrating (*i*) that proper mitochondrial functionalism relies on proper levels of mtDNA copies and (*ii*) the etiopathogenic cause of the dysfunction observed is because of NRTI toxicity and interference in mtDNA replication.

Under pathological conditions, mitochondrion triggers apoptosis. Consequently, any mitochondrial disarrangement may have fatal cell consequences. An important pre‐requisite for a successful pregnancy is that the maternal immune system does not reject the foetus and thus, cellular immune response could be essential. Apoptosis has also been shown to play an important role in promoting maternal immune tolerance to paternal antigens expressed by trophoblastic cells 45, which is a physiological process during pregnancy. While apoptosis is thought to be important as a normal physiological feature for foetal or placental development, enhanced levels may also be involved in the pathological conditions. Higher apoptosis levels may have implications in adverse perinatal outcome. A greater incidence of apoptosis has been observed in conditions such as pre‐eclampsia and IUGR, suggesting that appropriate regulation of apoptosis is important for normal pregnancy 46.

Blood cells apoptotic activation of caspase‐3 increased along pregnancy in both our cohort of HIV‐patients under treatment and in controls. In uninfected pregnancies the apoptotic rate of caspase‐3 activation probably increases as a physiological mechanism to delete newborn cells from the maternal blood 45. However, this increase was significantly enhanced in HIV pregnancies in concordance with accumulated, previous NRTI exposure. Apoptosis of uninfected cells is a key element of HIV pathogenesis and is believed to be the driving force behind the selective depletion of CD4^+^ T cells leading to immunodeficiency. We found that HIV infection and ARV have a significant impact on apoptotic activation of PBMC's caspases. In the first trimester of gestation HIV‐positive women showed higher levels of PBMC apoptotic caspase‐3 activation specially in those with longer exposure to HIV or NRTI. Additionally, at delivery, HIV‐infected women longer exposed to NRTI showed higher levels of PBMC caspase‐3 activation. These results are in agreement with previously published work 38.

However, we did not find any association between mtDNA content, mitochondrial protein synthesis, mitochondrial function or apoptotic caspase activation and adverse perinatal outcome in HIV patients. We found an increased prevalence of adverse pregnancy events in HIV pregnancies and enhanced trends towards mitochondrial impairment and apoptotic caspase activation along pregnancy in accordance to HIV or NRTI exposure, but did not find a significant association between these molecular findings and poor obstetric outcome. We did not find the presence of different patterns of mitochondrial toxicity and apoptotic caspase activation in HIV pregnancies correlating with a distinct pattern of clinical expression, probably because of the small sample size, which made patient stratification and statistical findings difficult. Other constraints of this study may be the presence of different types of HAART and time‐exposure to HIV or ARVs, characteristic of observational studies and personalized treatment interventions, which on the other hand may enclose findings to reality. The inclusion of a control group of non‐pregnant HIV‐infected and treated women may have been useful to assess longitudinal mitochondrial and apoptotic toxicity of HIV and HAART without potential gestation interference. However, such studies are extensively documented in the bibliography 47 and, additionally, the interest of the present work was focused on obstetric problems and, thus, in pregnant women. Additionally, to overcome methodological pitfalls and strength, the significance of reported findings, the deregulation of bioenergetics capacity or apoptotic status in studied patients was assessed, in parallel, by Western Blotting and functional measures, which rendered similar results. However, detailed mechanistic pathways underlying HIV‐ and HAART‐associated toxicity in HIV pregnancies should be further elucidated.

In conclusion, although pathogenetically plausible with these findings, it is not yet possible to prove a cause–effect association of adverse perinatal outcome with mitochondrial and apoptotic toxicity of HIV and NRTIs exposure during pregnancy. As a consequence, we did not succeed in the secondary objective of this study; identify epidemiological risk factors and prognostic markers of mitochondrial toxicity or apoptotic caspase activation for potentially associated poor clinical results in HIV‐infected mothers.

Several groups have proposed that monitoring possible markers of mitochondrial dysfunction in peripheral blood of pregnant women may be useful for detecting preclinical NRTI toxicity. Furthermore, prospective studies in HIV pregnancies under HAART are needed, to determine whether the incidence of mitochondrial disorders differs according to the regimen used and to develop predictive models to identify mothers–infants at highest risk. In an era of expanding treatment options, minimizing toxicities is possible and necessary. The role of mitochondria and apoptosis in physiological conditions must also be clarified to define the relevance of mitochondrial or apoptotic alterations in HIV pregnancies.

As a proof‐of‐concept, the present study has been conducted in a single‐site centre and in a limited population. The short‐ and long‐term health consequences of mitochondrial toxicity and apoptosis in HIV pregnancies should be further investigated in larger cohorts. There is a crucial need to fully understand the scope and depth of this problem through continued basic and clinical research evaluating the effects of foetal and maternal ARV exposure to better understand the morbidity associated with mitochondrial toxicity or apoptosis in pregnant women exposed to HIV and HAART. The current challenge is to design new ARV schedules with reduced harmful mitochondrial and apoptotic effects.

## Conflict of interest

None of the above mentioned authors have any financial, consultant, institutional and other relationship that might lead to bias or a conflict of interest for the information contained on the present manuscript.

## Supporting information


**Figure S1** Western Blotting results of COXII, COXIV, Caspase3 and β‐actin in HIV‐infected and treated or uninfected pregnant women (patients and controls, respectively) at first trimester of gestation (1T) and at delivery (D).

**Table S1** Raw data of mitochondrial DNA, protein synthesis, mitochondrial respiratory chain activity of complex II+III and apoptotic rate of caspase‐3 activation in HIV‐infected and treated or uninfected pregnant women.
