# Supplementary material for: Mitochondrial toxicity and caspase activation in HIV pregnant women
Source: J Cell Mol Med. 2016 Aug 30;21(1):26–34. doi: 10.1111/jcmm.12935 (PMC5192803; doi:10.1111/jcmm.12935)
Supplement: Supplementary file 1 — Figure S1 Western Blotting results of COXII, COXIV, Caspase3 and β‐actin in HIV‐infected and treated or uninfected pregnant women (patients and controls, respectively) at first trimester of gestation (1T) and at delivery (D). [file JCMM-21-26-s001.docx]

**1T**

**D**

**COX-II (15 KDa)**

**COX-IV (25.6 KDa)**

**1T**

**D**

**β-Actin (42 KDa)**

**Casp3(17-19 KDa)**

**Patient**

**Control**

**1T**

**D**

**1T**

**D**

**Patient**

**Control**

**Supplementary Figure1. Western Blotting results of COXII, COXIV, Caspase3 and β-actin in HIV-infected and treated or uninfected pregnant women (patients and controls, respectively) at first trimester of gestation (1T) and at delivery (D).**
