# Supplementary material for: Mitochondrial toxicity and caspase activation in HIV pregnant women
Source: J Cell Mol Med. 2016 Aug 30;21(1):26–34. doi: 10.1111/jcmm.12935 (PMC5192803; doi:10.1111/jcmm.12935)
Supplement: Supplementary file 2 — Table S1 Raw data of mitochondrial DNA, protein synthesis, mitochondrial respiratory chain activity of complex II+III and apoptotic rate of caspase‐3 activation in HIV‐infected and treated or uninfected pregnant women. [file JCMM-21-26-s002.docx]

**SUPPLEMETARY DATA**

**Supplementary Table1. Raw data of mitochondrial DNA, protein synthesis, mitochondrial respiratory chain activity of complex II+III and apoptotic rate of caspase-3 activation in HIV-infected and treated or uninfected pregnant women.**

|  | **HIV-infected and treated pregnant women** | | | | **Uninfected pregnant women** | | | |
| --- | --- | --- | --- | --- | --- | --- | --- | --- |
|  | **First Trimester**^a^ | **Delivery**^a^ | **% of increase or decrease along pregnancy** | **p** | **First Trimester**^a^ | **Delivery**^a^ | **% of increase or decrease along pregnancy** | **p** |
| **Mt DNA** | 5.72±0.62 | 3.28±0.34 | -42.66±5.94 | p<0.01 | 4.69±0.69 | 3.83±0.55 | -18.34±11.73 | NS |
| **Mt Protein synthesis (COXII/IV ratio)** | 1.17±0.07 | 1.02±0.07 | -12.82±5.73 | p<0.01 | 1.28±0.14 | 1.2±0.14 | -6.25±11.25 | NS |
| **Mt respiratory chain activity of complex II+III** | 25.85±3.01 | 20.55±2.62 | -20.50±10.14 | p<0.01 | 28.02±2.69 | 26.16±2.91 | -6.64±10.39 | NS |
| **Apoptotic caspase-3 activation** | 0.19±0.05 | 0.38±0.09 | 100±47.37 | p<0.001 | 0.11±0.04 | 0.18±0.05 | 63.64±45.45 | p<0.001 |

NS: not significant; Mt: mitochondrial

^a^ Data are presented as means ± standard error of the mean.


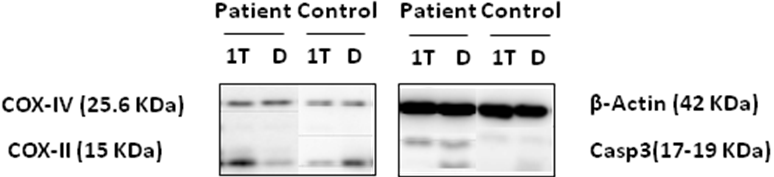


**Supplementary Figure1. Western Blotting results of COXII, COXIV, Caspase3 and β-actin in HIV-infected and treated or uninfected pregnant women (patients and controls, respectively) at first trimester of gestation (1T) and at delivery (D).**
